# Optical Glucose Sensors Based on Chitosan-Capped ZnS-Doped Mn Nanomaterials

**DOI:** 10.3390/s23052841

**Published:** 2023-03-06

**Authors:** Son Hai Nguyen, Phan Kim Thi Vu, Hung Manh Nguyen, Mai Thi Tran

**Affiliations:** 1 School of Mechanical Engineering, Hanoi University of Science and Technology, Hanoi 100000, Vietnam; 2 College of Engineering and Computer Science, VinUniversity, Hanoi 100000, Vietnam; 3 VinUni-Illinois Smart Health Center, VinUniversity, Hanoi 100000, Vietnam

**Keywords:** chitosan, glucose, sensor, ZnS-doped Mn, optical biosensor, UV–Vis, photoluminescence

## Abstract

The primary goal of glucose sensing at the point of care is to identify glucose concentrations within the diabetes range. However, lower glucose levels also pose a severe health risk. In this paper, we propose quick, simple, and reliable glucose sensors based on the absorption and photoluminescence spectra of chitosan-capped ZnS-doped Mn nanomaterials in the range of 0.125 to 0.636 mM glucose corresponding to 2.3 mg/dL to 11.4 mg/dL. The detection limit was 0.125 mM (or 2.3 mg/dL), much lower than the hypoglycemia level of 70 mg/dL (or 3.9 mM). Chitosan-capped ZnS-doped Mn nanomaterials retain their optical properties while improving sensor stability. This study reports for the first time how the sensors’ efficacy was affected by chitosan content from 0.75 to 1.5 wt.%. The results showed that 1 %wt chitosan-capped ZnS-doped Mn is the most-sensitive, -selective, and -stable material. We also put the biosensor through its paces with glucose in phosphate-buffered saline. In the same range of 0.125 to 0.636 mM, the sensors-based chitosan-coated ZnS-doped Mn had a better sensitivity than the working water environment.

## 1. Introduction

Although glucose is an energy source, having too much or too little in the blood might have adverse health effects. While excessive blood glucose levels result in diabetic disease, extremely low blood sugar can have serious health consequences, including seizures, stroke, and brain damage [1]. As a result, the healthcare system must monitor glucose levels and detect anomalies [2]. The industry-standard method for estimating glucose concentration in saliva using optical measuring tools is liquid chromatography–mass spectrometry (LC–MS) [3]. However, the process is time-consuming and requires expensive chemicals, specialized equipment, and trained personnel. Recently, optical biosensors based on nanomaterials have been developed to take the place of traditional glucose measurement methods [4]. Optical biosensors based on absorption and fluorescence detection appeal to researchers due to their low production costs, low specimen consumption requirements, low energy consumption, excellent accuracy, and rapid response. A variety of photosensitive materials, in particular semiconductors, were chosen to detect glucose based on its fluorescence intensity [5,6,7]. The optical properties of semiconductor nanoparticles were commonly modified using size-selective production and doping nanocrystals with the appropriate transition metal ions [8]. However, some of these compounds have the severe disadvantage of harming people. The main contributors to their cytotoxicity are heavy metal ion disintegration and release and highly reactive oxygen species [9]. On the other hand, several non-toxic materials are utilized in blood glucose testing, such as Mn-doped CdTe/ZnS [10]. Fluorescence resonance energy transfer sensors based on CdSe-ZnS quantum dots were reported by Freeman et al. to detect glucose with a limit of detection of 1.8 mg dL${}^{-1}$ [11]. To detect glucose in blood samples as low as 25 mg dL${}^{-1}$, Yu et al. introduced glucose oxidase (GOx) optical biosensors [12]. As an alternative, Zhang et al. described employing Pt electrodes coated with GOx/GNP/SWNT/PAA to create real-time enzyme glucose sensors for saliva samples [3]. Among all the semiconductor nanomaterials recently investigated in medicine, ZnS and ZnS nanocrystals doped with transition metal ions such as Cu${}^{2+}$, Co${}^{2+}$, and Mn${}^{2+}$ primarily exhibit properties such as water solubility, biocompatibility, high luminescence, image stability, and especially, low cytotoxicity. Because, at room temperature, ZnS has a bandgap energy of about 3.7 eV, making it transparent to solar spectrum wavelengths, fascinatingly, ZnS emission becomes more refined when doped with additional ionic transition metals such as Ni, Fe, Mg, Co, Mn, and Cu [13,14,15]. When combined with Mn${}^{2+}$ ions, ZnS nanoparticles emit light at 585 nm [16]. The energy transfer from the ZnS band gap to the Mn${}^{2+}$ dopant (from the triplet state 4T${}^{1}$ to the ground state 6A${}^{1}$) of the Mn${}^{2+}$ incorporated into the ZnS host lattice is what causes this phosphorescence emission [17].

Despite having improved luminescence properties, ZnS-doped compounds containing various transition metal ions frequently exhibit issues such as poor nanostructure quality and limited control over the emission color [17]. The production of ZnS-doped Mn using capped chitosan (CH) was recently described by Sharma et al. [17]. Since chitosan is hydrophilic, biodegradable, biocompatible, antigen-free, non-toxic, and biofunctional, it is the ideal polymer for biological applications [18]. In this context, chitosan stabilizes the ZnS nanoparticles (NPs) and alters the sample’s photoluminescence (PL) intensity and emission wavelength [19]. Additionally, GOx is the industry standard enzyme for bio probes due to its improved glucose selectivity. In particular, GOx is more resistant to extreme temperatures, pH levels, and ionic strengths than most other enzymes. As a result, making and affixing biological probes to semiconductor nanoparticles is relatively straightforward [20].

In this study, we prepared nanomaterials from ZnS-doped Mn with varying chitosan ratios (0.75%, 1%, 1.25%, and 1.5 %wt). Then, utilizing optical methods in a low testing range from 0.125 to 0.636 mM, the produced materials for GOx-based sensors were used to detect pure glucose and glucose in phosphate-buffered saline (PBS). We used PBS, regarded as a common buffer solution, to assess the biosensor’s selectivity. By estimating the sensitivity of ZnS-doped Mn with chitosan and GOx, our sensors’ high sensitivity, dependability, and quick response to a deficient glucose level were demonstrated. This work paves the way for the development of a diagnostic technology based on a small saliva sample that can deliver quick results with high reliability for clinical examination of patients and treatment outcome forecasting.

## 2. Materials and Methods

### 2.1. Chemicals

In our experiments, the raw materials used without any further purification for preparing the samples were zinc acetate (Zn (CH${}_{3}$COO)${}_{2}$·2H${}_{2}$O) (99.99%, Merck KGaA, Darmstadt, Germany), sodium sulphide nonahydrate (H${}_{19}$Na${}_{2}$O${}_{9}$S) (99.99%, Shanghai Zhanyun Chemical Co., Ltd., Shanghai, China), chitosan (C${}_{56}$H${}_{103}$N${}_{9}$O${}_{39}$) (99.99%, Shanghai Zhanyun Chemical Co., Ltd., Shanghai, China), manganese acetate (Mn (CH${}_{3}$COO)${}_{2}$·2H${}_{2}$O) (99.99%, Merck KGaA, Darmstadt, Germany), acetic acid (CH${}_{3}$COOH) (100%, Merck KGaA, Darmstadt, Germany), and distilled water.

### 2.2. Preparation of ZnS: Doped Mn Nanomaterials Capped with Chitosan (CH-ZnS/Mn)

First, we mixed 8.78 g zinc acetate and 0.08 g manganese acetate into 80 mL deionized water for 30 min at room temperature to make Suspension A. Meanwhile, Suspension B was created by completely dissolving 0.5 g chitosan (CH) in 40 mL acetic acid 1%. Suspension B was combined with Suspension A to make a final mixture with CH ratios of 0.75%, 1%, 1.25%, and 1.5 %wt. The 2.4 g sodium sulfide was then gradually added to the mixture and mixed for two hours. Precipitation began almost immediately, and the concentration of the residue increased. The mixture was transferred to a Teflon autoclave and incubated at $80{\;}^{\circ }$C for two hours. Following the hydrothermal treatment, the precipitation was cleaned several times by centrifuging it for five minutes at 4000 rpm with ethanol. Finally, the cleaned granules were dried in a vacuum oven for eight hours at $60{\;}^{\circ }$C.

### 2.3. Measuring the Optical Properties of Glucose Sensors Based on CH-ZnS/Mn

The optical absorbance of the produced CH-ZnS/Mn in the 220–800 nm range was measured using a DeNovix UV–Visible spectrometer (Model: DS-11 FX+). The samples were placed in 10 mm-thick cuvettes. The cuvettes were filled with 1500 μL of 1000 mg/L ZnS-doped Mn solution before gradually adding 0.2 mM glucose solution with volumes ranging from 100 μL to 700 μL. All samples’ photoluminescence (PL) characteristics were evaluated using a 10 nm slit-width spectrophotometer (SpectraPro HRS-300, Teledyne Princeton Instruments, Trenton, NJ 08619 USA). As previously stated, each sample was placed in a 10 mm-thick cuvette before being added.

## 3. Results and Discussion

### 3.1. Characterizations of CH-Capped ZnS-Doped Mn Nanomaterials

**Figure 1:**
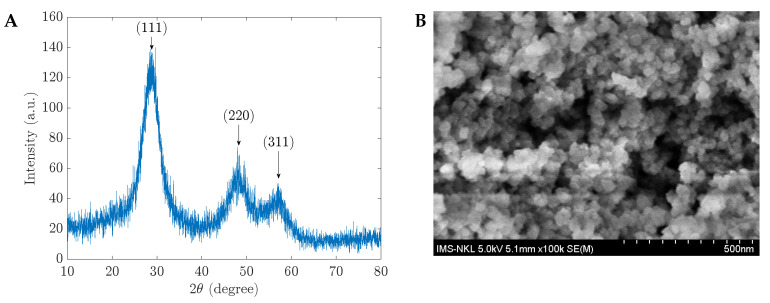
(**A**) XRD spectra and (**B**) SEM image of the 1 %wt CH-capped ZnS-doped Mn.

The produced materials were measured using the XRD and SEM images as the initial stage in characterizing their structure and shape. The 1 %wt CH-ZnS/Mn materials’ XRD and SEM results are displayed in Figure 1. Similar XRD spectrum patterns can be seen in the other materials with various CH percentages. In Figure 1A, three peaks at positions (111), (220), and (311) demonstrated the cubic sphalerite structure of ZnS-doped Mn (ZnS/Mn) (JCPDS Card No.5-0566). This observation indicates that the %wt CH may not significantly impact the structure of the starting components. The particles in those manufactured materials are shaped. The diameter of the grains is less than 500 nm (see Figure 1B). In the next section, the prepared materials were utilized in glucose sensing applications by UV–Vis measurements.

### 3.2. UV–Vis Measurements of Glucose Sensors Based on CH-ZnS/Mn Materials

**Figure 2:**
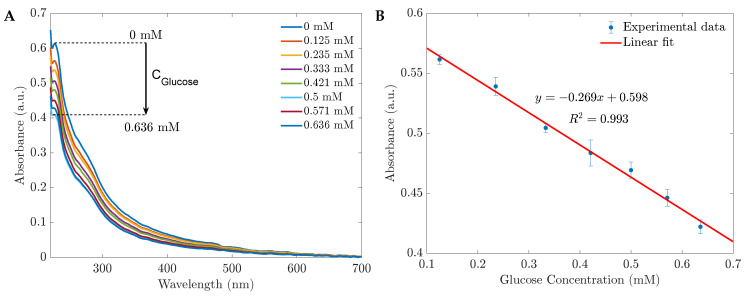
(**A**) The absorbance spectra of 1 %wt CH-ZnS/Mn 1000 mg/L reduce with the glucose concentration increasing; (**B**) the calibration curve of the glucose sensor within the range of 0.125 to 0.636 mM based on the absorbances at the peak of 230 nm.

**Figure 3:**
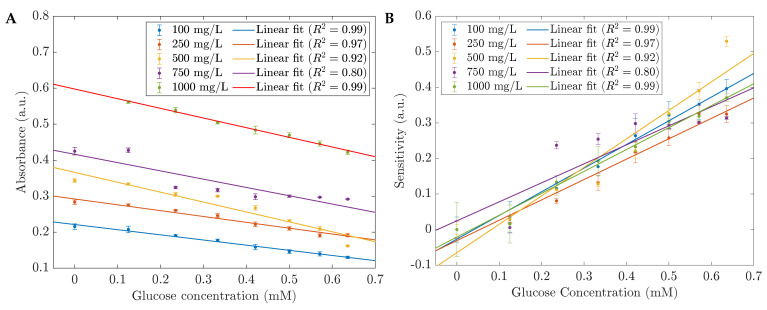
(**A**) The absorbances at the peak of 230 nm and (**B**) the sensitivities depend on the concentration of the sensing materials, 1 %wt CH-ZnS/Mn.

The UV spectra of 1 %wt CH-ZnS/Mn nanoparticles and their reactions to various glucose concentrations in water are shown in Figure 2A. The quenching effect of glucose is visible in the spectra. The biosensor’s absorbance decreased without a peak shift when the glucose concentration increased. In addition, the absorbance varied linearly with the glucose level in the range of 0.125 mM to 0.636 mM based on the absorbance at 230 nm (see Figure 2B). Figure 3 demonstrates the sensors’ performances with different CH-ZnS/Mn concentrations. The relationships between the raw absorbance at 230 nm and the sensitivity at 230 nm versus the glucose concentration are shown in Figure 3A,B, respectively. In Figure 3B, the sensitivity was calculated by the equation $S=\left(A_{0}-A\right)/A_{0}$, where *S* is the sensitivity. $A_{0}$ and *A* are the absorbances before and after adding glucose, respectively. Because of its high absorbance and high sensitivity (the highest precision) shown in Figure 3, 1000 mg/L of 1 %wt CH-ZnS/Mn was chosen for the absorbance glucose sensors’ fabrication.

### 3.3. Photoluminescent Measurements of Glucose Sensors Based on CH-ZnS/Mn Materials

**Figure 4:**
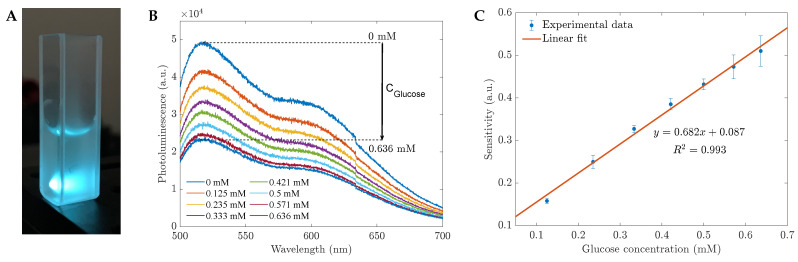
(**A**) The PL experiment setup; (**B**) the photoluminescence and (**C**) the sensitivity depend on the glucose concentration in water using the 1 %wt CH-capped ZnS-doped Mn sensing material based on the PL intensity at 530 nm.

In this section, we investigated the potential use of CH-ZnS/Mn nanomaterials for room-temperature photoluminescence (RTP) and employed photoluminescent (PL) measurements as an analytical analysis for the glucose biosensors. As shown in Figure 4, the quenching effect of glucose in water was observed. Based on the photoluminescent intensity, we can develop a functional operating system for TRP glucose sensors and calculate the sensitivity (*S*) corresponding to the glucose concentration by the equation $S=\left(I_{0}-I\right)/I_{0}$, where $I_{0}$ is the photoluminescence intensity before adding glucose and *I* is the corresponding intensity in contact with glucose. We examined the same region using UV–Vis measurements between 0.125 and 0.636 mM. The relationship between the intensity in this linear working range and the glucose levels can be described by the linear function y=0.682x+0.087. This result was determined from the average of 15 measurements.

### 3.4. CH-ZnS/Mn-Based Glucose Sensor Performance for Glucose in the Artificial Saliva and Phosphate-Buffered Saline and Its Sensitivity

**Figure 5:**
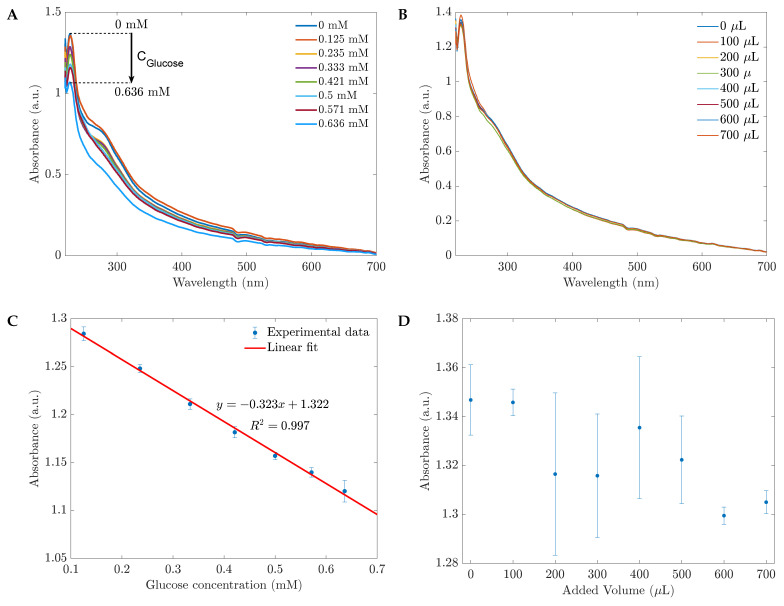
The absorption spectra of the sensor exposed to different concentrations of glucose in PBS (**A**) and the added volume of PBS (**B**). Absorbances of CH-ZnS/Mn-based sensors in response to (**C**) glucose in PBS and (**D**) only PBS based on the absorbances at 230 nm in (**A**,**B**), respectively.

**Figure 6:**
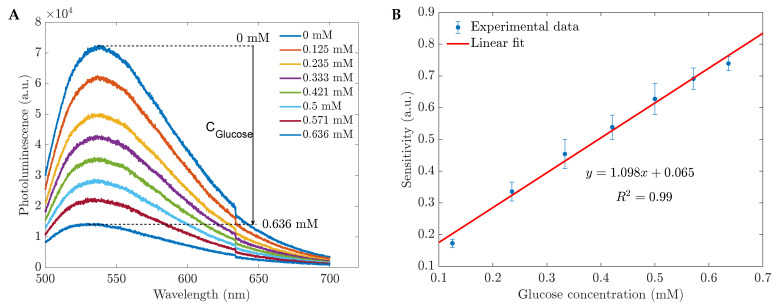
(**A**) PL spectra of the glucose sensors in PBS and (**B**) the sensitivity vs. glucose concentration calculated by PL intensities at 530 nm from (**A**).

In the previous section, the performance of the sensors for glucose in water was analyzed. Here, we examined the operating performance of the CH-ZnS/Mn sensor for glucose in phosphate-buffered saline (PBS) using UV–Vis and PL measurements. With glucose in PBS and PBS separately, the procedures in Section 3.2 and Section 3.3 were repeated. Our experiments showed that the UV spectra (Figure 5A) and PL measurements (Figure 6A) produced strong quenching signals in reaction to the glucose in the PBS. As a result of our observations, calibration lines that plot the sensitivity or the absorbance/PL against the glucose concentration can be developed. In particular, the calibration line for UV–Vis performance can be estimated as y=−0.323x+1.322, where *x* is the glucose concentration and *y* is the absorbance (see Figure 5C). As shown in Figure 5B,D, when the sensors and PBS (without glucose) were combined, the added volume changed from 100 μL to 700 μL, but the absorbance barely changed, and the trend of the change was not linear. This result demonstrated that glucose, not PBS, was responsible for the effects on the CH-ZnS/Mn sensors.

As we introduced the glucose in the PBS suspension, the emission spectra were captured at an excitation wavelength of 365 nm and are displayed in Figure 6A. When the glucose concentration increased, the absorbance and photoluminescence dropped, demonstrating agreement between the absorbance and emission spectra. When ZnS-doped Mn with a 1 %wt CH cap responded to the glucose in the PBS, the emission peaked at about 530 nm (Figure 6A). The quenching effect was represented in a calibration curve for the biosensor’s operating range of 0.125 to 0.636 mM (Figure 6B). After adding the glucose, the position of the emission peak remained constant. The sensitivity changed linearly with the concentration of the added glucose as a function of y=1.098x+0.065, where *x* is the glucose concentration (mM) and *y* is the sensitivity. With this high sensitivity and accuracy, the promising applications in clinical examinations of the fluorescent glucose sensors based on 1 %wt CH-ZnS/Mn were confirmed. Comparing the synthesized sensors to earlier works, Table 1 shows that they not only had a low limit of detection (LOD) and a low cost, but also a straightforward measurement setup.

### 3.5. The Effect of %wt CH on the Biosensor’s Performance

**Figure 7:**
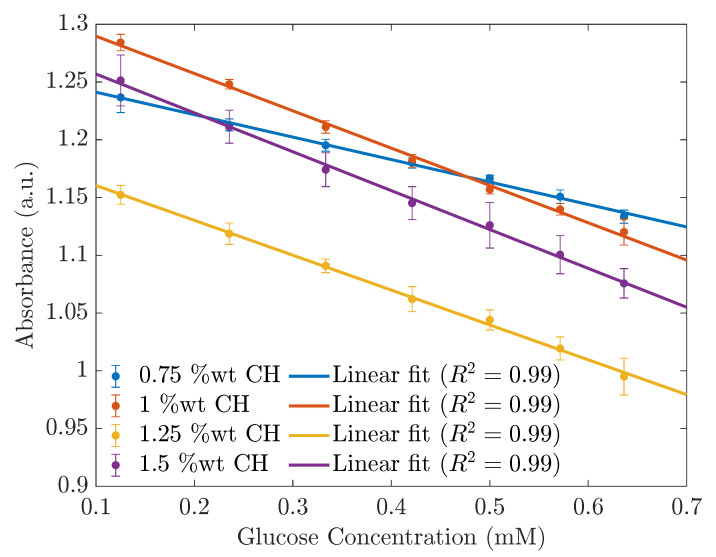
The calibration lines based on the absorbances at 230 nm depend on the concentration of the glucose in the PBS with different CH ratios.

**Figure 8:**
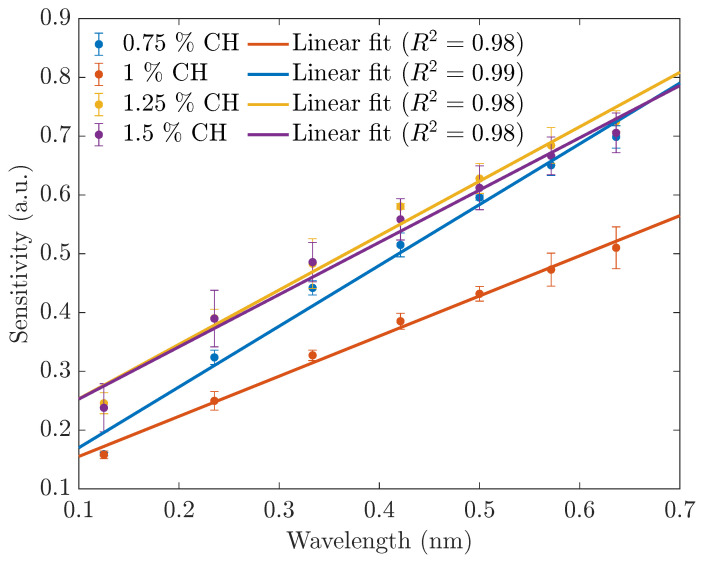
The sensitivities of the RTP glucose sensors in water with different CH ratios.

To investigate the effect of the %wt CH in the glucose sensing application of the ZnS/Mn-based sensors, we prepared four materials with the CH ratios of 0.75 wt%, 1 wt%, 1.25 wt%, and 1.5 wt%. Each material was suspended at a concentration of 1000 mg/L and tested for the UV and PL performances with different glucose concentrations. As shown in Figure 7, except for the 0.75 %wt CH-ZnS/Mn sensors, the rest of the sensors had similar slopes. However, the 1 %wt CH materials had the highest absorbance. This observation was also seen in the PL spectra (Figure 8), where the 1 %wt CH showed the most-linear and highest photoluminescence intensity. Hence, we recommend using the 1 %wt CH-ZnS/Mn for glucose sensing applications.

## 4. Conclusions

As a result of this investigation, we successfully manufactured CH-ZnS/Mn nanomaterials for glucose biosensors that performed significantly linearly and consistently in both water and PBS in the range of 0.125 mM to 0.636 mM. The developed sensor has a limit of detection of 0.125 mM. As per our recommendation, 1000 mg/L of 1 %wt CH-ZnS/Mn is the optimal sensor for glucose in water and PBS. In the following steps, we can test our sensors using various samples, such as glucose in artificial saliva, natural saliva, blood, or urine. Additionally, as mentioned in a recent publication, scientists employed CH-ZnS/Mn nanomaterials for various biological applications, including detecting bacteria [25], selenite [26], and other applications. We can optimize the materials developed for multiple purposes to create dependable, quickly reacting, and affordable gadgets.

## Figures and Tables

**Table 1:** Glucose sensors based on the optical approaches.

Biosensor Material	Detection Techniques	Linear Range (mM)	LOD (mM)	Ref.
Ag NP-GOx	Surface-enhanced Raman scattering (SERS)	2.0–14.0	2.0	[21]
GO@SiO_2_@Ag NPs@MPBA	Surface-enhanced Raman scattering (SERS)	2.0–20.0	2.0	[22]
Hydrogel fiber	Optical	0.0–20.0	NA	[23]
Au film-GOx	Optical fiber surface plasmon resonance (SPR)	0.0–2.8	NA	[24]
CH-ZnS/Mn-GOx	Absorbance/fluorescence quenching	0.125–0.636	0.125	Our work

## Data Availability

Not applicable.
